# Rad51 strand invasion function is needed for its role in CCTG tetranucleotide DNA repeat contractions

**DOI:** 10.17912/micropub.biology.002241

**Published:** 2026-08-10

**Authors:** Symphony A. Rutkowski, Neila V. Serumaga, Eva J. Bond, Jane C. Kim

**Affiliations:** 1 Biological Sciences, Cal State San Marcos, San Marcos, CA, United States; 2 Charlie Dunlop School of Biological Sciences, UC Irvine, Irvine, CA, United States

## Abstract

Myotonic Dystrophy Type 2 is a genetic disorder caused by expanded CCTG DNA repeats in the *CNBP* gene. By using a genetic assay in *Saccharomyces cerevisiae* and knocking out *RAD51* , we previously found that homologous recombination is involved in large-scale CCTG repeat contractions. In this study, we measured contraction rates using the *RAD51-II3A* allele, which is defective in homology search and strand invasion. We found that the mean contraction rate in the *RAD51-II3A* strain was indistinguishable from *rad51Δ* , indicating that structural properties alone of the repetitive CCTG sequence cannot initiate double strand break repair by promoting strand invasion.

**Figure 1. Large-scale contractions of (CCTG) 100 repeats are reduced in the RAD51-II3A yeast strain with a mean rate indistinguishable from rad51Δ:** (A) Model of large-scale CCTG repeat contractions in *Saccharomyces cerevisiae* , which is proposed based on previous genetic analysis (Papp et al. 2024). An expanded tract of CCTG/CAGG repeats results in elevated double strand break formation, which may be mediated by the repeats’ secondary structure-forming capabilities. Following strand unwinding by a specialized helicase and 5’ to 3’ end resection (enzymes not depicted), Rad51 (purple diamond) will be loaded on the 3’ single-stranded DNA tail(s). “Out-of-register” repeat misalignment towards the distal end of the repeat tract could result in a large-scale contraction, which is feasible because of the repetitive nature of the invading strand and homologous template. (B) Experimental system to study large-scale CCTG repeat contractions *in vivo* (Papp et al. 2024). (C) Mean rate of large-scale contraction for (CCTG) _100_ strains based on at least five independent experiments, shown with standard error. Individual rates were calculated using the Ma–Sandri–Sarkar maximum-likelihood estimator with a correction for sampling and plating efficiency, which was based on the number of Ura+ clones in 8-12 cultures grown in parallel, per experiment (at least n=5 per genotype). The mean rates for the *rad51Δ* and *RAD51-II3A* strains are significantly lower than wild type (* p<0.0001) and not significantly different from one another.

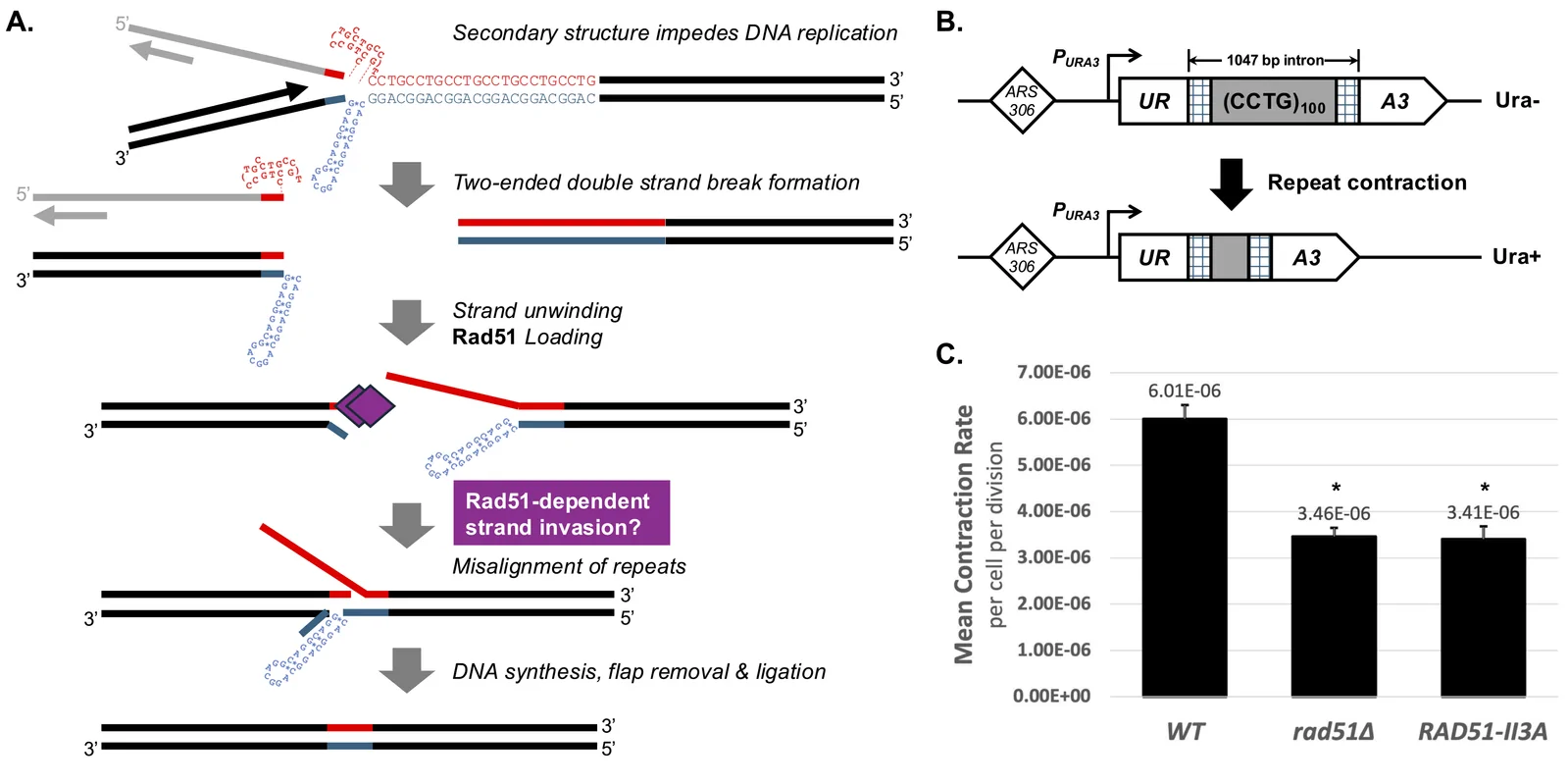



## Description

Repeat expansion diseases are a group of over 40 inherited conditions where the causal mutation is an abnormally long tract of short tandem repeats within a specific gene. These DNA repeats, or microsatellites, can range in length from three to twelve nucleotides, though the majority associated with human diseases are trinucleotide repeats. Myotonic Dystrophy Type 2 (DM2) is a neuromuscular disorder caused by expansion of CCTG tetranucleotide repeats in the first intron of *CNBP* , a gene that encodes CCHC-type zinc finger nucleic acid binding protein. In unaffected individuals, the CCTG repeat tract is typically fewer than 30 repeats, whereas up to ~11,000 repeats have been observed in DM2 patients (Liquori et al. 2001). Accumulation of the CCUG RNA results in toxicity stemming from gain-of-function effects, though other mechanisms such as repeat-associated non-AUG translation may also contribute to disease pathology (Marzullo et al. 2026). Various studies have investigated trinucleotide repeat instability and shown that DNA replication, repair, and recombination are, under various conditions, involved in repeat expansions and contractions (Khristich and Mirkin 2020). However, the molecular pathways that modulate CCTG repeat instability are not as well-studied.

Using a chromosomal arm loss assay, we previously showed that *Saccharomyces cerevisiae* strains with (CCTG) _100_ showed elevated DNA breakage (Papp et al. 2024). Furthermore, we found that homologous recombination (HR) was important for large-scale CCTG contractions, as knocking out critical components of HR such as *RAD51* and *RAD52* significantly decreased contraction rates. HR is a high-fidelity DNA repair mechanism that utilizes a homologous sequence as a template to repair double-strand breaks (DSBs) and support replication fork recovery (Symington et al. 2014). Rad51, the main recombinase involved in HR, assembles into a nucleoprotein filament on single-stranded DNA and facilitates the search for homologous sequences and strand exchange.

We previously described a model ( Figure 1A ) where, following repeat-induced DSB formation at the replication fork, Rad51 and Rad52 promote DNA strand invasion of the CCTG 3′ overhang to the homologous template (Papp et al. 2024). The model depicts the newly displaced long CAGG tail forming a hairpin structure, which is based on *in vitro* evidence that the CAGG orientation forms a more stable hairpin than the CCTG orientation (Dere et al. 2004). If strand invasion occurs “out-of-register”, this could result in a large-scale contraction. We proposed that these events happen during S phase since treatment with either camptothecin or hydroxyurea, drugs that cause replication stress, elevated large-scale contraction rates. There was no difference in contraction rates between wild type and *rad59Δ* strains, indicating that the mechanism is not canonical single strand annealing. Furthermore, though replication strand slippage on the template strand may contribute to some repeat contractions, we proposed that these are likely to be deletion events of several repeat units rather than the 80 repeat contractions we observed.

This study is designed to test the hypothesis that the strand invasion function of Rad51 is required for its role in large-scale CCTG contraction events. An alternative hypothesis is that sequence-dependent annealing of CCTG and CAGG strands can entirely substitute for the strand invasion function of Rad51 in this system, which is based on the unique biophysical properties of the repeats. Specifically, nuclear magnetic resonance studies using oligonucleotides with short CCTG or reverse complementary CAGG repeats (Lam et al. 2011; Guo and Lam 2016) showed that CCTG repeats form secondary structures such as hairpins and dumbbells that are fluid and dynamic, displaying an ability to change between different conformations and shift along the repetitive tract. Consequently, the fluid secondary structures might allow a CCTG 3′ overhang to promote the search for DNA homology and invade the double-stranded repetitive template without the strand invasion function of Rad51.

To distinguish between the two hypotheses described above, we used CRISPR gene editing to construct the *RAD51-II3A* allele, which retains DNA-binding ability *in vitro* but is defective in catalyzing strand exchange and is defective in mitotic HR *in vivo* (Cloud et al. 2012). This enabled us to investigate how Rad51 strand invasion activity contributes to the instability of CCTG tetranucleotide repeats using our previously described genetic assay for large-scale contractions ( Figure 1B ) (Papp et al. 2024). In this system, (CCTG) _100_ repeats are located in the artificial intron of a *URA3* reporter gene. The reporter gene is integrated ~1 kb downstream of the replication origin *ARS306* . The starting strain with (CCTG) _100_ is Ura-. Repeat contraction renders the cells Ura+, and the frequency of these mutant clones can be used to calculate a rate of contraction. Importantly, only contractions greater than ~80 repeats will be selected in this assay.

We evaluated the rate of large-scale CCTG contractions in yeast strains with the (CCTG) _100_ reporter and either the wild type, *rad51Δ* , or *RAD51-II3A* backgrounds. We observed a mean contraction rate of 6.01 × 10⁻⁶ (± 3.02 × 10⁻⁷ SE) in the WT (n = 6 trials). In *rad51Δ* , the mean contraction rate was 3.46 × 10⁻⁶ (± 1.87 × 10⁻⁷) for n = 6 trials, and in *RAD51-II3A* it was 3.41 × 10⁻⁶ (± 2.73 × 10⁻⁷) for n = 5 trials. Compared to wild type, this represents ~1.7-fold reduction in contraction frequency for *rad51Δ* and *RAD51-II3A* ( Figure 1C ). The contraction rate analysis was statistically significant compared to wild type (p = 0.0000153 for *rad51Δ* ; p = 0.0000745 for *RAD51-II3A* , one-tailed unpaired t-test). There was no significant difference between *rad51Δ* and *RAD51-II3A* strains on the rate of large-scale CCTG repeat contractions.

The similarity in contraction rates between *rad51Δ* and *RAD51-II3A* strains indicates that the strand invasion function of Rad51 is needed for Rad51-dependent contraction events. This finding aligns with the observation that Rad51 is required not only for DSB repair at non-repetitive regions but also for recombination-mediated resolution of complex DNA structures (Kerrest et al. 2009). Although the role of HR had been well-established for trinucleotide repeats, the current study highlights that HR and Rad51-dependent strand invasion is similarly important for the instability of tetranucleotide DNA repeat sequences. Future studies will continue to investigate the precise genetic control of CCTG and reverse complementary CAGG repeat expansions, contractions, and fragility.

## Methods

**Yeast strains:** The *RAD51-II3A* allele encodes three alanine substitutions (R188A, K361A, K371A) in site II of Rad51. Whereas site I binds resected single-stranded DNA with high affinity, site II contains a basic patch that promotes its interaction with homologous double-stranded DNA (Cloud et al. 2012). This allele was introduced into YJK168 (Papp et al. 2024), a yeast strain that enables selection of large-scale contraction of (CCTG) _100_ within a *URA3* reporter gene, using CRISPR gene editing. We used CRISPR plasmid p73 (Papp et al. 2024) that targets *RAD51* and a repair template amplified via PCR from a previously described *RAD51-II3A* yeast strain MT151 (Tsabar et al. 2015) for standard lithium acetate yeast transformation. Transformants were screened by PCR and restriction digest, as the *RAD51-II3A* allele introduces a new HhaI site, followed by Oxford Nanopore sequencing (Plasmidsaurus) of the PCR-amplified full-length gene. All strains were plated to select for plasmid loss and ensure that Cas9 endonuclease is no longer expressed.

**Fluctuation analysis to determine contraction rates:** Five independent trials were performed to determine contraction rate in the *RAD51-II3A* yeast strain (YJK398). Each trial comprised 12 distinct clones/colonies, which consisted of two independent isolates for each strain/genotype. After three days of growth at 30°C on YPD media supplemented with additional uracil (50 μg/mL), an individual colony was suspended in sterile water ( *e.g.* 500 μL). To select for Ura+ clones, 100 μL of the 10 ^⁻1^ dilution cell suspension was plated onto synthetic media lacking uracil (SC-URA). For each clone, 100 μL of the 10⁻⁵ dilution was plated onto non-selective YPD media to calculate the total number of viable cells in each culture. Colony counts from both SC-URA and YPD plates were recorded after 72 hours of incubation at 30 °C. Colonies that showed a change in repeat length, either contractions or expansions, based on PCR analysis were omitted from the dataset. The rate analysis was carried out using the web-based FluCalc tool (Radchenko et al. 2018). This method uses the Ma-Sandri–Sarkar maximum likelihood estimation (MSS-MLE) equations for calculating mutation rates (Sarkar et al. 1992). Statistical significance was evaluated by independent unpaired t test for single hypothesis testing.

## Reagents

**Yeast Strains**

**Table:** 

**Strain**	**Description**	**Source**
MT151	*hoΔ hml::ADE1, MATα hmr::ADE1 ade1-110, leu2,3-112, lys5, trp1::hisG, ura3-52, ade3::GAL:HO, RAD51-II3A::TRP1*	Tsabar et al
YJK168	*MATa, leu2-Δ1, trp1-Δ63, ura3–52, his3–200, ChrIII(75594-75641)::URA3-Int-(CCTG)100*	Papp et al
YJK272	YJK168; *rad51Δ*	Papp et al
YJK398	YJK168; *RAD51-II3A*	This study

**Primer Sequences**

**Table:** 

**Name**	**Sequence**	**Description**
JK544	TAGCGACAAAGAGCAGACGTAG	With JK686, amplify *RAD51-II3A*
JK686	CTTCATAGATCGCGAACACACATTCAGCtTCTGGTAAGCAAGGTGAGTCAACAACAGCGC	With JK544, amplify *RAD51-II3A* repair template (mutates NGG)
JK499	AAGACCGCAGTAGGGTTGCGAGG	With JK544, amplify complete gene for mutation verification
JK213	GTCCTGTGGATCCTCTACGC	Verify repeat length
JK214	GAGGTTATGGGAGAGTGAAAAATAG	Verify repeat length
